# Risky business: a scoping review for communicating results of predictive models between providers and patients

**DOI:** 10.1093/jamiaopen/ooab092

**Published:** 2021-11-12

**Authors:** Colin G Walsh, Mollie M McKillop, Patricia Lee, Joyce W Harris, Christopher Simpson, Laurie Lovett Novak

**Affiliations:** 1 Department of Biomedical Informatics, Vanderbilt University Medical Center, Nashville, Tennessee, USA; 2 Department of Medicine, Vanderbilt University Medical Center, Nashville, Tennessee, USA; 3 Department of Psychiatry and Behavioral Sciences, Vanderbilt University Medical Center, Nashville, Tennessee, USA; 4 Center for AI Research and Evaluation, IBM Watson Health, Cambridge, Massachusetts, USA; 5 Center for Knowledge Management, Vanderbilt University Medical Center, Nashville, Tennessee, USA

**Keywords:** predictive analytics, predictive algorithms, patient communication, risk communication, shared decision-making

## Abstract

**Objective:**

Given widespread excitement around predictive analytics and the proliferation of machine learning algorithms that predict outcomes, a key next step is understanding how this information is—or should be—communicated with patients.

**Materials and Methods:**

We conducted a scoping review informed by PRISMA-ScR guidelines to identify current knowledge and gaps in this domain.

**Results:**

Ten studies met inclusion criteria for full text review. The following topics were represented in the studies, some of which involved more than 1 topic: disease prevention (*N* = 5/10, 50%), treatment decisions (*N* = 5/10, 50%), medication harms reduction (*N* = 1/10, 10%), and presentation of cardiovascular risk information (*N* = 5/10, 50%). A single study included 6- and 12-month clinical outcome metrics.

**Discussion:**

As predictive models are increasingly published, marketed by industry, and implemented, this paucity of relevant research poses important gaps. Published studies identified the importance of (1) identifying the most effective source of information for patient communications; (2) contextualizing risk information and associated design elements based on users’ needs and problem areas; and (3) understanding potential impacts on risk factor modification and behavior change dependent on risk presentation.

**Conclusion:**

An opportunity remains for researchers and practitioners to share strategies for effective selection of predictive algorithms for clinical practice, approaches for educating clinicians and patients in effectively using predictive data, and new approaches for framing patient-provider communication in the era of artificial intelligence.

## INTRODUCTION

It appears to me a most excellent thing for the physician to cultivate Prognosis; for by foreseeing and foretelling, in the presence of the sick, the present, the past, and the future, and explaining the omissions which patients have been guilty of, he will be the more readily believed to be acquainted with the circumstances of the sick; so that men will have confidence to entrust themselves to such a physician.

—The Book of Prognostics, Hippocrates

Fundamental to caregiving, prognostication dates to the beginning of medicine. Clinical prognostication has evolved beyond patient and provider judgment alone to include the output of rules, risk scores, and increasingly complex predictive algorithms. Vast amounts of data from electronic health records (EHRs) and more recently direct-to-consumer genetic testing, self-tracking or wearable devices yield unprecedented opportunity for prognostication.^1–3^ But despite the fervor for new prognostic technology, operational implementation has not followed the pace of technology development. This leaves health care providers at the dawn of a proliferation of predictive technology, with little guidance on best practices in communicating the output of algorithmic prognoses to patients. In today’s health care environment, human-centered design methods are increasingly preferred for designing technology interventions that fit with workflow,^4^ especially when patients are potential consumers of the information.^5^^,^^6^ This review examines the available evidence to guide the design and implementation of predictive risk information sharing with patients.

## BACKGROUND AND SIGNIFICANCE

Traditionally, the therapeutic relationship between patients and care providers mediated the communication of poor prognoses. Today, novel algorithms provide risk estimates, recommendations around those estimates, and intent to improve or streamline decision-making. In the era of hype around healthcare artificial intelligence (AI), this information flow—prognosis by machine—can be scaled to health-system, national, or even international scale.^7^ Thus, knowledge gaps on how best to communicate such information will potentiate negative consequences of implementing AI into practice and erode trust in such systems among organizations, providers, and patients. An important subset of this area will be algorithmic results communicated directly to patients just as laboratory results and notes have been released through patient portals without human-in-the-loop approval or decision-making. If providers and organizations communicate effectively with patients about risk, patients will be more empowered for self-care and participation in treatment decisions.

PubMed systematic reviews of “predictive models” numbered 73 in 2010 and over 600 in 2020. Yet understanding of how algorithmic results are best communicated to patients requires similarly rigorous review of the literature. In this paper, we review the state of the literature around communication of results of predictive algorithms from providers and provider organizations to patients. Although direct-to-consumer prognostication is expanding, we focus on providers and provider organizations as this remains the primary way in which clinically relevant risk estimates are communicated to patients. The driving question is: *What is the best evidence around communication of algorithmic results by providers to patients?* Because of the diverse nature of the literature in this space, we structured our study as a scoping review.

### Concepts and definitions

The area of search relies on concepts that are currently not well-defined or are defined differently by discipline or domain. Therefore, we define key terms in Table  1.

**Table 1.** Definition of concepts included in search

Term	Definition
Risk	The provision of a value such as a likelihood, a probability, an expected class label or membership that would be conveyed to patients as a recommendation of future event(s) related to their health and healthcare.
Providers	Physicians and others who may be referred to as providers in the literature, including surgeons, nurses, social workers, and other allied health professionals. We also include institutional actors such as health care organizations and insurance companies.
Algorithms	A series of steps used to solve a problem. We will use “algorithm” to describe the object that results from those steps, often called a “model.”
Predictive analytics	The range of computational and statistical techniques to estimate likelihood of future events—for example, outcomes, disease onset, or severity, utilization, etc.
Communication	Direct communications between individual health care workers and patients, and communication between organizations (eg, health care providers, health plans) and patients. Communication includes conversations, electronic messages, letters, or other forms of information exchange such as decision aids and risk profiles. Communication research may include studies of comprehension and usability of various information representations for inclusion in the above types of communication.

We take a wide view of the definition of algorithm. Common active examples include the Atherosclerotic Cardiovascular Disease (ASCVD) Risk Calculator,^8^ Braden score for pressure ulcers in nursing,^9^ Framingham risk score,^10^ APACHE/APACHEII in the ICU, CHADS/CHADS2/CHADS2-Vasc,^11^^,^^12^ readmission risk scores,^13^ and sepsis prediction.^14^^,^^15^ These scores might be simple, manually computable by a physician, for example, “LACE”^16^ for readmissions), or complex, for example, deep neural networks. The inverse relationship between algorithmic interpretability and performance complicates this area.

For predictive analytics, we include both the concepts of prediction and prognosis to extend the scope of our search and focus on studies that problematized communication of predictive analytics results specifically. Yet, we acknowledge that within the context of health, prognosis and prediction have historically been differentiated. For example, a growing body of literature supports prognosis as a forecast in the absence of intervention versus a prediction which should include the potential impact of intervention in that same forecast.^17^

## MATERIALS AND METHODS

**Figure 1.**
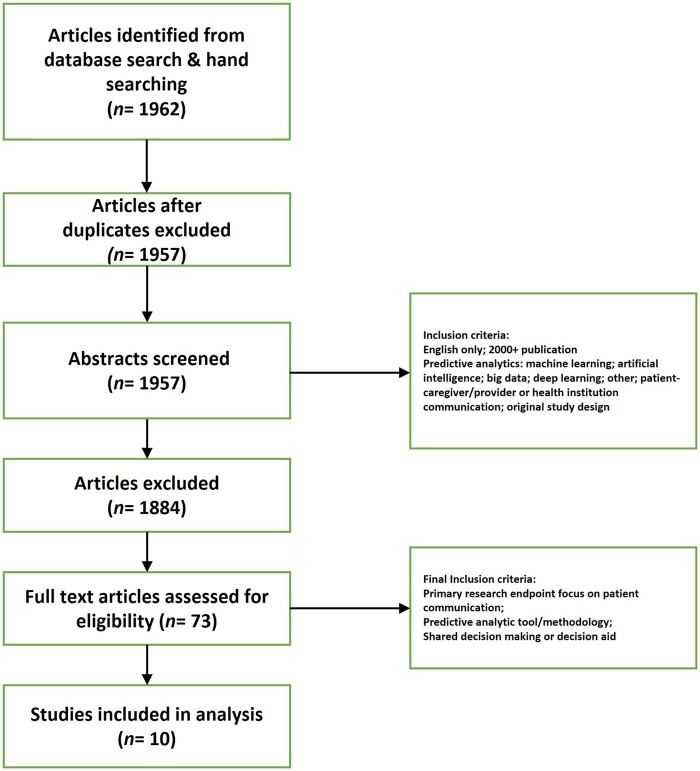
Search methodology.

Our methodology for a scoping review followed the PRISMA-ScR^18^ Statement. A medical information scientist (author PL) searched PubMed on November 12, 2019 with the rationale that it is the single best primary published source for peer-reviewed literature for both clinicians and bioinformaticians. We tested 3 distinct concepts in our search (1) communication; (2) predictive analytics, AI, deep learning, big data, machine learning, risk scoring; and (3) communication between the patient or caregiver and doctor/physician/health care provider or patient/caregiver and health system communication using Medical Subject Headings (MeSH) and nonstandard terms. Figure 1 depicts the methodology.

We conducted an initial search on September 9, 2019, and the updated final search on November 12, 2019. Broad results using “communication” in the initial search strategy required enrichment with additional words and phrases to improve recall in the final search (see Supplementary Material S1 for full search terms). Results were restricted to primary research only, publications in years 2000+, included English only, and eliminated some publication types (letter, news, comment, legal case). Editorials identified in hand searches were included to assist identification of seminal papers and relevant controversies. Studies focused solely on genetic risk counseling or interpretation were held out of scope as were studies centered on the ethics surrounding algorithmic bias. Other methods used to identify relevant material include: individual expertise of our team members, hand-searching and reviewing bibliographies of selected papers or systematic reviews. After abstract screening, 73 articles advanced to full text review.

Title and abstract screening were performed by authors LLN, CGW, JWH, MMM, and CS for eligibility for full text review by 2 blinded independent reviewers. If 2 reviewers did not agree, a pre-assigned third reviewer conducted blind adjudication. Studies were included for full text review if titles or abstracts indicated evaluation of communication of algorithmic results to patients. Final inclusion criteria required: a primary metric of patient communication; a predictive analytic tool; and a decision aid or tool for shared decision-making between patient and provider. The team used a REDCap^19^ database to conduct the screening and a system of shared folders for tracking the evaluated and classified studies. Each study was reviewed by 2 or more team members and presented to the rest of the team in group discussions. A qualitative synthesis process that involved multiple iterations of classification and discussion enabled the team to group the studies and identify the key contributions related to the research question. On final review the team documented the following elements from each paper: application area (ie, goal of the communication), disease focus, and population. Reviewers wrote a brief summary of each paper. Additional methodological details are found in the Supplementary File.

## FINDINGS

Ten articles met final inclusion criteria (Table  2).^20–29^ The studies were funded through government,^21–23^^,^^25–27^ foundation,^20^^,^^23^ university,^28^^,^^29^ and commercial^24^ sources. The articles represented diverse topic areas, but a limited number of studies examined communication related to patient-specific predictive results. By application area, most studies focused on disease prevention and treatment decisions. Five of 10 (50%) focused on disease prevention.^20^^,^^21^^,^^27–29^ Five (50%) focused on treatment decisions in various clinical scenarios: thrombolysis in acute stroke,^22^ prostate cancer therapy,^25^ chemotherapy in breast cancer,^26^ and cardiovascular disease (CVD).^24^^,^^27^ One (10%) focused on harms reduction through identification of potentially inappropriate medication usage.^23^ Of included studies, a subset included visualization or user interface examples of their tools.^21^^,^^22^^,^^25^^,^^29^ We summarize the studies below, grouped into areas of predictive risk application, and then describe several contribution areas that spanned multiple papers, providing a basis for assessing both the state of the science and the remaining gaps. Contribution areas were identified by the team through qualitative synthesis of the methods and findings of the papers.

**Table 2.** Summary of findings

Publication	Application area					
	Disease preventionaids	Treatment decision aids	Medication harms reduction	Disease focus	Population	Brief summary
Asimakopoulou et al^20^	✓			Cardio-vascular	Patients with type 2 diabetes, *n* = 95	A comprehension study to assess 3 timeframes for perceived, understood, and recalled risk representation (1, 5, and 10 years).
Bonner et al^21^	✓			Cardio-vascular	Primary care patients, *n* = 25	A design study on heuristics and biases observed when patients think aloud while interpreting various risk representations.
Flynn et al^22^		✓		Thrombolysis	Acute stroke patients and family members, *n* = 14	A usability study to assess various graphical risk representations.
Fried et al^23^			✓	No specific disease	Veterans over 65 years old prescribed 7 or more medications, *n* = 128	A controlled trial of the effect of a medication risk report on shared decision-making.
Grover et al^24^		✓		Cardio-vascular	Primary care patients, *n* = 3053	A controlled study to evaluate a graphical coronary risk profile.
Hakone et al^25^		✓		Cancer	Men with prostate cancer over 65 years old	An evaluation study of a decision-making tool with multiple risk and treatment scenarios.
Mühlbauer et al^26^		✓		Cancer	Breast cancer patients	A study to understand the impact of available online prediction tools used to create a novel, printed decision aid.
Persell et al^27^	✓	✓		Cardio-vascular	Adults, community health centers	A study to assess the potential benefits of statin prescribing through telephone and mailed outreach by lay health workers.
Sheridan et al^28^	✓			Cardio-vascular	Internal medicine clinic patients	A controlled intervention study to understand a tailored risk decision aid.
Skinner et al^29^	✓			Cancer	Primary care patients	A study to understand the impact of a computerized risk assessment tool in the clinic waiting room.

### Disease prevention decision aids

Sheridan et al^28^ randomized patients to receive or not receive a coronary heart disease (CHD) focused decision aid including “individualized information” about global CHD risk, patients’ specific risk factors, and preventive strategies to employ. Patients randomized to the intervention group used the “Heart to Heart” decision aid tool that provided 10-year risk information based on the Framingham equation as well as recommendations for CHD risk prevention, with the motivation being that allowing patients to assess their personal risk and weigh preferences would increase patient-provider communication. The control group received only a list of the patient’s CHD risk factors for presentation to their doctor. The intervention tool shares the pros and cons of risk-reducing therapies to help patients make their own decisions, and the risk reduction achievable after one or more therapeutic interventions to understand the additive benefit of risk reduction. It also encourages patients to choose therapies that are acceptable and feasible for long-term risk reduction. For example, the tool encourages a good diet and regular exercise for everyone. With regards to how this information is communicated, information is delivered on the computer but can also be printed-out to take to one's visit with their doctor. The intervention group seemed to be more likely to discuss CHD risk with their providers and to develop and to intend to follow specific preventive plans. The decision aid did not increase patients’ desire to participate in shared decision-making compared to controls.^28^

A comparison of probabilistic CVD risk calculators by Bonner et al included qualitative methods (“think aloud”) on patients’ preferences for presentation and formatting of CVD risk presentation in a general practice setting. Two different interfaces were used to display graphical information to participants. Key differences in risk communication that were tested included timeframe for risk (eg, 5 vs 10 years), graphical display of risk (eg, risk categories vs linear risk score), family history collected as part of presentation of individual risk, and effect of intervention risk factors that varied in presentation by how they were linked to individual risk and modifiability. One was embedded in an existing health management app and another through a publicly available website. Key findings included the importance of reference points to assist in interpretation of risk data, the credibility and novelty of risk scores, and participants’ selective attention to information. As an example of the latter category, patients not taking medications focused on potential side effects of medications and the small potential risk reductions compared to lifestyle interventions.^21^

Skinner et al studied the Cancer Risk Intake System (CRIS) and its impact on facilitation of patient-clinician discussion about tamoxifen, genetic counseling, and colonoscopy. CRIS generated printed recommendations based on family history, personal health history, existing medical conditions, and various other risk factors. Information was communicated to the patient through a stand-alone application running on a touch-screen tablet computer in the clinic waiting room prior to a clinic visit. CRIS supplies modular messages to convey risk information, selecting messages from a library of 162 potential messages with an average length of 125 words, up to 3 messages for inclusion in tailored printouts, which were shown as simple text-based paper printouts in the manuscript. The printout can then be used for discussion among participants and clinicians during appointments. Emphasis was placed on using nonmedical language and abbreviations and recommendations were framed as discussion points with one’s doctor, to avoid encouraging specific behavioral decisions. Based on the level of risk, the printouts contained recommendations for 1 or more of the following: breast cancer prevention via tamoxifen, genetic counseling, and/or colon cancer surveillance. Most participants reported that the CRIS system prompted discussion about tamoxifen, genetic counseling and colonoscopy.^29^

In Asimakopoulou et al,^20^ investigators studied the accuracy of perceived risk for stroke and CHD using the United Kingdom Prospective Diabetes Study Risk Engine tool, finding diabetic patients inflate their personal risk for CHD or stroke by self-report in either 1, 5, or 10 years using a 0–100 point scale. This implies that in communicating risk or treatment recommendations to CHD patients there is a need to account for this cognitive bias. The study used the United Kingdom Prospective Diabetes Study (UKPDS) Risk Engine, a T2D risk calculator for uncomplicated diabetes that estimates risk in different time frames for CHD and stroke to examine 3 types of CHD and stroke risk estimate characteristics: patients’ perception; understanding of actual risk after use of the risk engine and research nurse consultation; and memory for the risk 6 weeks later for all 3 time frames. Objective personalized data from the risk engine was first presented in a bar chart and percentages. The study supplemented risk engine estimates for 1, 5, and 10 years with sets of smiley faces and a 10-slice pie chart to graphically depict risk. The outcome metric using actual, perceived, and understood risk was measured in a 6-week recall for each of the 3 timeframes. Patient perceived, understood, and recalled risks varied 2–30 times greater than actual risk, finding that the risk tool plus decision aids corrected patient misperceptions of their personal risk of CHD and stroke.

### Treatment decision aids

Flynn et al studied a decision aid predicting outcomes with and without thrombolysis, including death and disability in acute stroke care. The decision aid, named COMPuterised decision Aid for Stroke thrombolysiS (COMPASS), was tested using paper and iPad representations of the tool and usability testing was performed. Risk was communicated in clustered and stacked bar graphs, pictographs, and flowchart diagrams. Graphical risk facilitated better understanding of the benefits and risks associated with thrombolysis among patients/relatives. Specifically, patients responded to risk information being presented in 1 view and real-time updates of risk estimates when patients changed risk information. The study also found the type of risk presentation (eg, pictograph) seemed to better convey probabilistic information consistent with research demonstrating acceptability in people with differing health literacy skills. This also helped to facilitate the acquisition of verbatim (specific probabilistic information) and gist knowledge (general impression). Some patients also had preference on the ordering of risk presentation preferring the order of independence, dependence, and death risk. Finally, the study highlights how the patient’s mental state impacts their ability to perceive risk, implying it is important to educate patients when they are not under extreme stress (such as just have received a very serious diagnosis).^22^

To study the effects of telephonic and mail outreach aimed at those eligible for but not receiving statins for primary prevention of CVD, Persell et al trialed lay health workers communicating with patients identified with CVD risk factors on the potential benefits of statin prescribing. Mail outreach consisted of individualized recommendations for participants, which were calculated using existing EHR data. The study emphasized the importance of highlighting individual risk and suggesting clinical actions along with risk estimates to facilitate discussions with one’s doctor. Telephonic outreach was performed by lay health workers. While the intervention group saw increased rates of discussion of cholesterol treatment, “most discussions did not result in the prescription of a statin.” Additionally, there was no significant difference between the intervention and control groups with respect to the clinical outcome, lower LDL-cholesterol within 1 year.^27^ This study included a disease prevention intervention, but is summarized only in this section for brevity.

Mühlbauer et al^26^ used Adjuvant! Online, PREDICT, and CancerMath, online prediction tools addressing the effect of adjuvant pharmacotherapy on survival, to inform the design of a novel decision aid for women with early hormone-receptor positive breast cancer. The decision aid included cancer risk data and individual prognosis information (eg, age, comorbidities). In showing risk of death, the authors found it may be important to distinguish risk of cancer death and risk of dying from other causes to support comprehension of risk from the disease itself. Additive effects of treatment are also important to communicate so that patients understand potential benefit. At the same time, it’s important that patient mental state and personal preferences are considered—too much information after receiving a diagnosis can be overwhelming and some patients did not want to see information about death. After pilot-testing using focus groups and an online survey testing numeracy and comprehension, results showed that researchers successfully communicated complex prognosis, treatment, and side effects information in an understandable manner. They acknowledged a digital, interactive decision aid tool might prove to be more user-friendly than the study’s print brochure format.

Hakone et al^25^ reported the results of an iterative design process for an online prostate-cancer risk-communication tool, named PROgnosis Assessment for Conservative Treatment (PROACT). The tool used two published clinical prediction models and iterative design to communicate patient risk and treatment options and measured comprehension. Here the goal of the tool was to constrain navigation so that the users view all aspects of the information in a structured narrative sequence to standardize conversations with their doctors. To determine this sequence, expert consensus among clinicians was used. Visualizations were also made interactive to encourage exploration of risk. The types of risk presented included: (1) survival ratio between dying from prostate cancer and overall survival rate, (2) comparison of survival rates between active vs. conservative treatment, and (3) temporal trend of cancer prognosis. To present each of these risks the authors experimented with a pie chart, which has been shown to be effective at depicting part-to-whole relationships, (4) a bar graph to compare treatment options, and (5) a temporal area chart to show patterns over time. Through patient interviews the research found emotional state at the time of receiving risk information is important and that patient sequence of thinking should also be incorporated into the sequence of how risk information is presented. Disease versus treatment should be presented separately, as risk must be understood first before treatments can be decided upon. Temporal visualization of disease progression for treatment decision making was also helpful in this process.

The CHECKUP study^24^ examined the impact of a CVD risk profile indicating probability of disease at baseline compared to usual care on patients. The intervention group patients received a graph in addition to the probability printout showing relative risk of a sample of matched adults for age and gender. Intervention patients had information on their own personal risk plus they were able to discern their absolute risk compared to the population sample. After the 3-month follow-up another risk profile was created showing changes after statin initiation and/or lifestyle modification. From the CHECKUP study, the researchers conclude that CVD risk was best presented to patients as percentages in visual formats such as graphs (bar charts and pictograms), as patients may inflate perceived risk compared to actual risk and graphical tools help to adjust this risk correctly. Patients in the intervention risk profile group were also more likely to reach lipid targets. The authors conclude presenting comparative risk plus personal level risk can lead to risk factor reduction, but the effect depended on the level of personal risk. If personal risk was high, “negative emotions are heightened, and comparative risk is attended to less.” The authors found that in presenting forecasts, “shorter timeframes (less than 10 years) may lead to more accurate risk perceptions and increased intention to change behavior.” They called for better quality trials to understand what risk presentation formats were best for achieving behavior change, and that less attention should be paid to the accuracy of risk prediction compared to effective communication and presentation of risk.

### Medication harms reduction

To reduce inappropriate medication prescribing at the Veterans Affairs Medical Center,^23^ Fried et al evaluated the Tool to Reduce Inappropriate Medications (TRIM). Using data comprising of medication and chronic disease data from EHRs with manual chart review and telephonic assessment, an algorithm was developed to identify medication reconciliation issues and potentially inappropriate medications and regimens among patients at the VA. TRIM provides feedback via online reports to both the patient and clinician regarding inappropriate medication prescriptions. The algorithm generates a short report for the patient that lists medication reconciliation discrepancies and reported problems with medications. This report is given to the patient just before a clinic visit to discuss medication concerns with the clinician. In a small trial, TRIM facilitated clinician communication, medication-related communication between physicians and patients, and correction of medication discrepancies. TRIM had no effect on the number of medications prescribed or reduction in potentially inappropriate medications.

### Key contributions

We identified several themes that crossed application areas, including source of the communication, contextualization of the results, and visualization.

Regarding source, effective patient-provider communication of algorithmic results might not originate with the provider. In Persell et al^27^ and Fried et al,^23^ active outreach across multiple channels and personnel catalyzed more discussion of treatment options though did not change prescription rates.^23^^,^^27^ Bonner et al^21^ delivered CVD risk estimates directly to patients either through an app or online.

Contextualizing risk information added to perceived credibility, understanding, and satisfaction with model output in Bonner et al,^21^ Flynn et al,^22^ and Mühlbauer et al.^26^ Mechanisms for this improvement included overcoming cognitive bias and attending to health literacy, acuity, and timeframe of predicted outcome. For example, paper-based solutions to disseminate complex algorithmic results showed benefit when used in context of health literacy and shared decision-making in families.^22^ In Bonner et al,^21^ time was a relevant contextual factor; longer time frames helped participants identify the need to take action for the highest risk groups, but shorter time frames were easier to plan for and more relevant to people who were older. Data from Askimakopolou^20^ showed investigators testing risk understanding among 3-time frames: 1, 5, and 10-year risk; patients self-report was more accurate for shorter time frames (1 and 5 vs 10 years). Flynn et al^22^ stressed the importance of time dependency in contextualizing estimates for predicting thrombolysis outcomes.

Data visualization is an essential element of contextualization. Several methods were used to examine the effectiveness and acceptability of visualization options, including usability interviews^25^ and quantitative assessment of comprehension.^20^^,^^24^ Flynn et al^22^ described pictograms to convey probabilistic information, consistent with research that describes their acceptability in people with lower literacy.

## DISCUSSION

**Figure 2.**
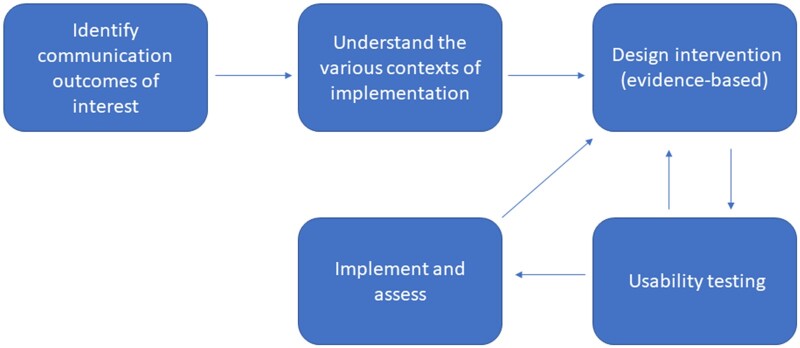
Human-centered design framing for research in patient-provider communication of predictive analytic results.

Primary findings of this review highlight a diverse but limited corpus covering a range of application areas. Much of medical practice involves predicting prognosis, and the body of literature on patient-provider communication is extensive.^30–33^ But we found few studies that explicitly discussed both together. Prognostic decision aids increased communication between patients and providers, yet data on measurable behavior change and downstream impact were mixed. We found studies involving user feedback on various visualizations, but most lacked documentation of specific impacts of design decisions. These research gaps and the heterogeneity of reviewed literature highlight a lack of standardization of human-centered design concepts and heuristics^4^^,^^34^ related to predictive analytics and communication of their results. We find few studies describe the way results are communicated explicitly. For example, it is unclear across studies the conditions under which results should be communicated such as the impact of the patient’s emotional and clinical context to understand risk. Finally, few studies described how representation of risk impacted measured outcomes. Our results highlight the following themes in need of further, more rigorous study: source of communication and communication context, and impact of design choices, which together lend themselves to a user-centered design framing (Figure  2).

### Source of communication and communication context

The studies we identified raised questions about the optimal source of predictive risk information for example, from the health care organization via an app, or in the context of a visit with a healthcare professional. Research examining patient-provider communication that includes *discussion* of risk communication results would also be enlightening, particularly research that identifies distinctions based on clinical context such as prognosis and patient emotional state, and practice setting for example, primary care vs. specialty. The shared decision-making domain^35–37^ includes useful frameworks for such investigations. Several papers in this study referred to or used shared decision-making^22^^,^^23^^,^^28^ as a framework.

### Design choices

Design choices impacted findings and merit further attention. Other research has shown that pictograms facilitated improvement in acquisition of verbatim (specific probabilistic information) and gist knowledge (general impression),^38^ and that in general, diagrams can facilitate problem-solving through spatial location of information.^39^ Bonner et al referenced 11 consensus-derived key components of risk communication from Trevena et al which include “(1) presenting the chance an event will occur; (2) presenting changes in numeric outcomes; (3) outcome estimates for test and screening decisions; (4) numeric estimates in context and with evaluative labels; (5) conveying uncertainty; (6) visual formats; (7) tailoring estimates; (8) formats for understanding outcomes over time; (9) narrative methods for conveying the chance of an event; (10) important skills for understanding numerical estimates; and (11) interactive web-based formats.”^38^^,^^40^

While the themes above are topical and timely, the evidence identified in our review is also preliminary. The disparate nature of qualitative and quantitative results in the included studies defied rigorous meta-analysis. Critical emphasis on clinically meaningful outcomes, for example, cardiovascular mortality, as opposed to proximal steps in the causal or therapeutic pathway, for example, discussion of statin prescription, will significantly add to the rigor and impact of studies like these in the future. Better justification of the decision-making and conceptual framework that led to design and implementation of studied solutions would strengthen future studies in this domain. A consensus statement on research frameworks and methods would assist the research community in producing findings with impact. Finally, it must be noted that most of the decision aids in our study were designed to facilitate discussion with one’s provider. Physician understanding of risk results is also fundamental to patient understanding and decision making. Current evidence shows even physicians have difficulty in understanding predictive analytic output.^41^ Although provider ability to understand predictive risk models as an element of communication was not in scope for this study, it is an important area for future research.

This review has implications in multiple domains including communications, quality, and standards research. First, few studies adhered to accepted or validated frameworks of communication^42^ which diminishes potential rigor and generalizability. Second, studies lacked standard reporting metrics preventing meta-analytic consideration. Third, as models proliferate in the literature and in operations, lack of guidance and standards related to communicating their results to patients will be increasingly difficult to attain without community effort. As predictive models are also monetized and aggregated datasets commercialized, such guidance becomes paramount to avoid the proliferation of poor or untested informatics strategies.

Several topics relevant to clinical decision support were not well-represented in our scoping review but merit mention. First, our understanding of risk also should evolve to incorporate novel and often rapidly evolving knowledge. For example, how should we incorporate clinical markers of risk gleaned from traditional data with non-clinical markers of risk, for example, environmental, psychosocial, genetic, in our communication with patients? Moreover, how does a holistic representation of risk best incorporate the effects of environment, behavior and activity, nutrition, financial factors, and more into effective decision support? Terminology standardization and knowledge curation might help address these questions. As models mature they must also be reevaluated for model and calibration drift.^43^^,^^44^ The literature in this area has grown, but we found no research on how patients or providers might interpret results in light of model dynamism and drift.

While out of scope here, ethical, legal, social, and privacy concerns must remain the subjects of future research. Hospitals’ use of predictive models challenge traditional understanding of “informed consent.”^45^ Similarly, non-disclosed patient characteristics might be rediscovered through computational means. Algorithmic bias as well as lack of transparency complicate analysis and reporting of sensitive healthcare data.^7^^,^^46^ While employing predictive algorithms and communicating results to patients may be well-intentioned, future research should aim to understand the impacts and unintended consequences, such as amplifying existing health inequities, as a result of their use.

We recommend several steps that can be taken to address the knowledge gaps identified in this review:

- Where possible, enhance predictive analytics projects to include studies of provider (organizations or clinical personnel) communication with patients.
- In patient-provider communication research on predictive analytics, adopt rigorous theoretical frameworks and report findings in ways that are accessible to meta-analysis.
- Study the relationship between patient-provider communication strategies and patient outcomes.
- Develop a framework for ethical considerations related to sharing predictive data with patients.
- Develop user interface design patterns that could be used in various contexts for patient-provider communication about predictive analytics results.

## CONCLUSION

Despite the growing number of published and marketed predictive models in healthcare, best practices for how to communicate the results of predictive models with patients have not emerged. We identified themes and implications across multiple domains for both research and operations in this scoping review. A human-centered design framework provides a structured way to consider potential research contributions to improve the evidence for effective patient-provider communication of predictive analytics results.

## FUNDING

This research was conducted jointly by Vanderbilt University Medical Center and IBM Watson Health with financial support from IBM Corporation.

## AUTHOR CONTRIBUTIONS

CGW, LLN, MMM, and PL conceived of the research questions, PL conducted the literature searches and facilitated the review process. CGW, LLN, MMM, CS, and JWH reviewed abstracts and evaluated full-text articles. All authors contributed to writing and editing and approved the final manuscript.

## SUPPLEMENTARY MATERIAL

Supplementary material is available at *JAMIA Open* online.

## Supplementary Material

ooab092_Supplementary_Data
