# Supplementary material for: Risky business: a scoping review for communicating results of predictive models between providers and patients
Source: JAMIA Open. 2021 Nov 12;4(4):ooab092. doi: 10.1093/jamiaopen/ooab092 (PMC8598291; doi:10.1093/jamiaopen/ooab092)

Walsh et al “Risky business: a scoping review and plan of action for communicating results of predictive models between providers and patients**”**

Supplementary File

Table of Contents

PUBMED Search Terms……………………………………………………………………………………………………………………………2

PRISMA-ScR Checklist………………………………………………………………………………………………………………………………3

Legacy PubMed Search Strategy

Retrieval limited to January 1, 2000 – November 12, 2019

(((((("doctor-patient"[tiab] OR "doctor patient"[tiab] OR "physician-patient"[tiab] OR "physician patient"[tiab] OR "patient-provider"[tiab] OR "patient provider"[tiab] OR "patient"[tiab] OR "provider-patient"[tiab] OR "provider patient"[tiab] OR "health system"[tiab] OR "health insurance"[tiab] OR "Caregivers"[Mesh] OR "caregiver"[tiab] OR "caregivers"[tiab] OR "caregiving"[tiab] OR "caretaker"[tiab] OR "Physician-Patient Relations"[Mesh] OR ("patients and providers"[tw]))) AND (("Communication"[MeSH] OR "communication barrier"[tiab] OR "communication barriers"[tiab] OR "speech perception"[tiab] OR "persuasive communication"[tiab] OR "health communication"[tiab] OR "clinical communication"[tiab]))) AND ((("predictive analytics"[tiab] OR "big data"[Mesh] OR "big data"[tiab] OR "artificial intelligence"[Mesh] OR "artificial intelligence"[tiab] OR "machine learning"[tiab] OR "machine learning"[Mesh] OR "deep learning"[tiab] OR "natural language processing"[tiab] OR "NLP"[tiab] OR "neural network*"[tiab] OR "risk scoring"[tiab] OR "risk score"[tiab] OR "risk prediction"[tiab] OR "risk assessment"[tw] OR "risk detection"[tiab] OR "early warning"[tiab] OR "clinical decision"[tw] OR algorithm*[tw] OR "forecasting"[mesh])))) AND ("2000/01/01"[PDat] : "2019/11/12"[PDat]) AND English[lang])

NOT

(systematic[sb] OR review[ptyp] OR Case Reports[ptyp] OR Comment[sb] OR interview[ptyp] OR patient education handout[ptyp] OR lecture[ptyp] OR bibliography[ptyp] OR meta analysis[ptyp] OR Interactive Tutorial[ptyp] OR Legal Case[ptyp] OR Interview[ptyp] OR Legislation[ptyp] OR Letter[ptyp] OR News[ptyp] OR Newspaper Article[ptyp]))

NOT (((("Communication"[Mesh]) AND (("doctor-patient"[tiab] OR "doctor patient"[tiab] OR "physician-patient"[tiab] OR "physician patient"[tiab] OR "patient-provider"[tiab] OR "patient provider"[tiab] OR patient[tiab] OR "provider-patient"[tiab] OR "provider patient" OR "health system"[tiab] OR health insurance[tiab]) OR Physician-Patient Relations[Mesh]))) AND ("predictive analytics"[tiab] OR "big data"[Mesh] OR "big data"[tiab] OR "artificial intelligence"[Mesh] OR "artificial intelligence"[tiab] OR "machine learning"[tiab] OR machine learning[Mesh] OR "deep learning"[tiab] OR "natural language processing"[tiab] OR "NLP"[tiab] OR "neural network*"[tiab] OR "risk scoring"[tiab] OR "risk score"[tiab] OR "risk prediction"[tiab]) AND (("1900/01/01"[PDat] : "2019/11/12"[PDat]) AND English[lang])) AND (("1900/01/01"[PDat] : "2019/11/12"[PDat]) AND English[lang])


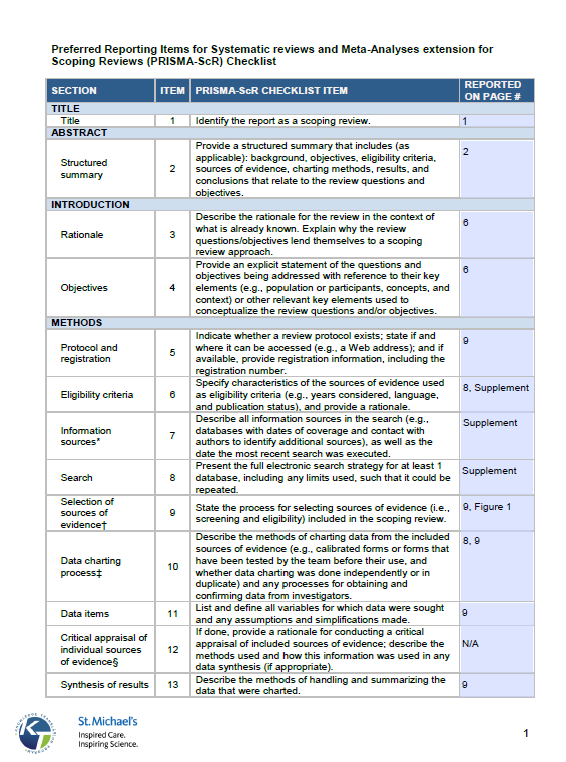


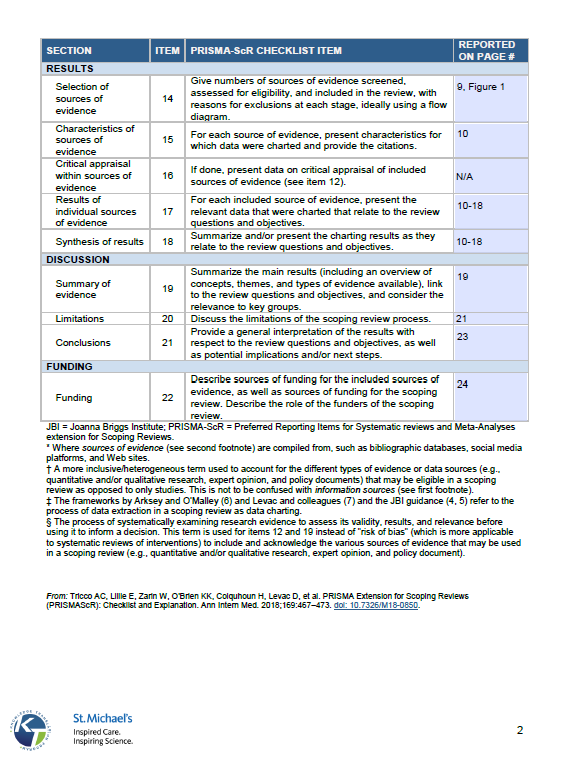

Supplement: ooab092_Supplementary_Data [file ooab092_supplementary_data.docx]
